# NO-HYPE: a novel hydrodynamic phantom for the evaluation of MRI flow measurements

**DOI:** 10.1007/s11517-021-02390-2

**Published:** 2021-08-08

**Authors:** Giacomo Gadda, Sirio Cocozza, Mauro Gambaccini, Angelo Taibi, Enrico Tedeschi, Paolo Zamboni, Giuseppe Palma

**Affiliations:** 1 Section of Ferrara, National Institute for Nuclear Physics (INFN), 44122 Ferrara, Italy; 2 Department of Advanced Biomedical Sciences, University of Naples “Federico II”, 80131 Napoli, Italy; 3 Department of Physics and Earth Sciences, University of Ferrara, 44122 Ferrara, Italy; 4 Vascular Diseases Center - Translational Surgery Unit, University of Ferrara, 44124 Ferrara, Italy; 5 Institute of Biostructures and Bioimaging, National Research Council, 80145 Napoli, Italy

**Keywords:** Phase-contrast MRI, Human circulation, Blood flow simulation, Hydrodynamic phantom

## Abstract

**Abstract:**

Accurate and reproducible measurement of blood flow profile is very important in many clinical investigations for diagnosing cardiovascular disorders. Given that many factors could affect human circulation, and several parameters must be set to properly evaluate blood flows with phase-contrast techniques, we developed an MRI-compatible hydrodynamic phantom to simulate different physiological blood flows. The phantom included a programmable hydraulic pump connected to a series of pipes immersed in a solution mimicking human soft tissues, with a blood-mimicking fluid flowing in the pipes. The pump is able to shape and control the flow by driving a piston through a dedicated software. Periodic waveforms are used as input to the pump to move the fluid into the pipes, with synchronization of the MRI sequences to the flow waveforms. A dedicated software is used to extract and analyze flow data from magnitude and phase images. The match between the nominal and the measured flows was assessed, and the scope of phantom variables useful for a reliable calibration of an MRI system was accordingly defined. Results showed that the NO-HYPE phantom is a valuable tool for the assessment of MRI scanners and sequence design for the MR evaluation of blood flows.

**Graphical abstract:**

**Figure:**
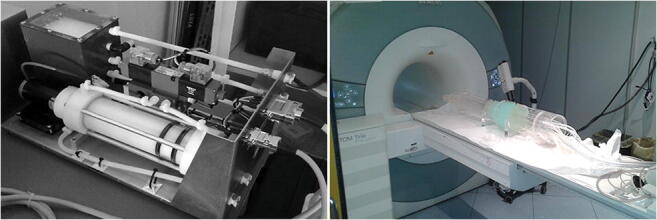
Overview of the NOvel HYdrodynamic Phantom for the Evaluation of MRI flow measurements (NO-HYPE). Left: internal of the CompuFlow 1000 MR pump unit. Right: Setting of the NO-HYPE before a MRI acquisition session. Soft tissue mimicking material is hosted in the central part of the phantom (light blue chamber). Glass pipes pass through the chamber carrying the blood mimicking fluid.

## Introduction

Quality control with a quantitative magnetic resonance imaging (MRI) phantom is necessary to ensure the accuracy and precision of results [1]. Indeed, system constancy data should be tracked regularly at all MRI systems and especially those used for quantitative measurements [2]. Measurement of blood flow velocity using phase-contrast (PC) MRI technique has been performed since the advent of MRI in the early 1980 [3], thus demonstrating a valuable non-invasive technique [4, 5] for both qualitative and quantitative assessment of flow [6–8].

Currently, the technique is benefiting from the incorporation of several technological advancements in MRI, such as increased gradient and field strengths, and higher signal-to-noise ratio (SNR) from multichannel coils. Also, time-resolved measurement of the blood flow velocity using cardiac-gated PC-MRI is gaining increasing interest in the clinical practice due to its capability to characterize the entire hemodynamic cycle [9]. Such technological improvements led in turn to the introduction of novel methods of data acquisition and analysis, which allow to derive additional information such as flow rate [10], pressure [11], and wall shear stress (WSS) [12].

The hemodynamic information from rapid PC-MRI scans is valuable in different clinical scenarios [13], but the accuracy of PC-MRI measurements is often unknown, thus limiting its actual incorporation in clinical practice. PC-MRI has been extensively studied in vivo in the aortic arch and carotid arteries [14–16], as well as in small structures such as cerebral arteries and intracranial aneurysms [17]. However, any accurate in vivo validation of velocity measurement is challenging, and an assessment of the technique through a dedicated phantom should be warranted beforehand, as exhaustively stated in the paper by Keenan et al. [2]. Such work reports that the Radiological Society of North America (RSNA), through the Quantitative Imaging Biomarkers Alliance (QIBA), promotes the development of quantitative imaging phantoms [18]. Efforts are also made by other institutions such as the American Association of Physicists in Medicine and the European Communities Biomedical Engineering Advisory Committee [19–22].

Given this background, in this study, we built and validated a flow phantom to understand, test, and optimize PC-MRI measurements for reliable clinical settings. The main contributions of the study are:

- To develop a MRI-compatible hydrodynamic phantom to simulate physiological blood flows
- To build the phantom and check the mechanical and electrical robustness of the pumping system
- To test the phantom in a clinical setup for the first time
- To collect and analyze the data acquired from the MRI scanner

To highlight the aforementioned contributions, the Methods section is divided in subsections describing:

- The hydraulic pump for blood flow simulation
- The design of the hydrodynamic phantom
- The flow data acquisition and analysis

The Results section is divided in subsections that report and analyze simulations of:

- Constant flow
- Pulsatile flow:
  - Sine flow
  - Physiologic flow

## Methods

1.1 *Hydraulic pump for blood flow simulation*

**Fig. 1:**
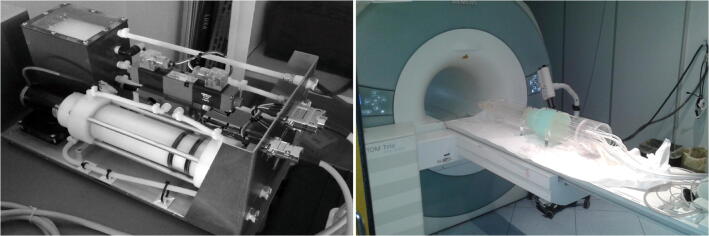
Left: CompuFlow 1000 MR (internal of the pump unit). Right: setting of the hydrodynamic phantom before a MRI acquisition session. Soft tissue-mimicking material is hosted in the central part of the phantom (light blue chamber). Glass pipes pass through the chamber carrying the blood mimicking fluid

The CompuFlow 1000 MR (Shelley Medical Imaging Technologies) [23] is a positive displacement pump designed for researches on simulated blood flow [24]. It is a two unit system consisting of a control assembly unit and a pump assembly unit (Fig. 1, left) that allows to dispense fluid at precise and accurate steady and pulsatile flow rates.

The piston is driven on a precise lead screw by a motion controller, programmed to eject the appropriate volume of fluid at a predetermined time interval. The resulting flow is accurate within ± 1% over a range of 0.1–35 ml/s. By interchanging the outlet and inlet paths when the piston reaches the end of its travel, a nearly uninterrupted output flow is achieved, thanks to a 4-port directional flow control valve; the pump refills one side of the cylinder, meanwhile it pumps fluid out the other side. A dedicated software allows to run the pump and perform both constant and pulsatile flow rates of the desired waveform. Such flow waveforms are synchronized with the MRI sequences through a custom, in-house developed, pulse oximeter device simulating the heartbeat, which is used as input to the MRI scanner for the retrospective gating of the signal acquisitions. The designed waveforms are provided as a file that contains the data points sampling the waveform (normalized between − 1 and + 1), the scale value that defines the peak value, and the time interval between the points. As the pump is not MR-compatible, it must be placed outside the MRI room.

1.2 *Design of the hydrodynamic phantom*

The MR-compatible phantom was connected to the pump outside the MRI room through long polyvinyl chloride (PVC) connections, which also ensure laminar flow and avoid turbulence. The phantom is made of 12 straight and parallel glass pipes of variable diameters (ranging from (9.0 ± 0.1) mm to (15.0 ± 0.1) mm) located into a bicylindrical plexiglass case (Fig. 1, right).

The length of each glass pipe, as well as the distance between hydraulic connectors, is 1000 ±1 mm. The lengths of the two plexiglass cylinders are 460 ± 1 mm and 440 ± 1 mm, while the diameters are 196 ± 1 mm and 146 ± 1 mm, respectively. The two cylinders are coaxial and fixed together at the bases to form the protective case for the glass pipes. In the central part of the case, an isolated chamber (length (440 ± 1) mm) contains the soft tissue-mimicking liquid crossed by the glass pipes. A large number of tissue-mimicking materials are described in the literature, created using different techniques [25–29]. The material used in this experiment is a water solution of 10 mmol per liter of CuSO_4_ [30]. The ion concentration of CuSO_4_ in water leads to relaxation time values similar to the ones of human soft tissues at clinical magnetic field intensities. Such mixture is cheap and easy to be produced in-house, thus allowing to perform several tests at different CuSO_4_ concentration before filling the phantom. We are aware that solutions can change its properties in time. For example, its homogeneity could change, thus compromising the tissue-mimicking property with respect to MRI. We performed the presented and other acquisitions in several weeks, and we never observed any significant change in the MR image quality. Therefore, we can state that the solution is stable enough to allow reliable measurements in different acquisition sessions. The blood-mimicking fluid (BMF) was produced by Shelley Medical Imaging Technologies and simulates the physical characteristics of blood for MRI studies (see Table 1) [31].

1.3 *Flow data acquisition and analysis*

**Table 1:** MRI properties of the used BMF. The manufacturer provides information about 1.5 T magnetic field only [31]

T_1_	850 ms
T_2_	170 ms
Density	1.02 g/cm^3^
Viscosity	4.1 mPa*·*s

The acquisitions were performed on a 3 T scanner (Siemens Trio, Siemens Medical Systems, Erlangen, Germany). The pumping system was set to produce both constant and pulsatile flows, with amplitudes and frequencies selected to span a range of physiological interest (the complete list of the acquisition schemes is reported in Table 2).

**Table 2:** List of acquisitions performed

Flow type	Q [ml/s]	VENC [cm/s]	f [Hz
Constant	5	50	/
	5	75	/
	10	50	/
	10	75	/
Sine	5	50	1
	10	50	1
	15	50	1
	10	50	0.3
	10	50	0.5
	10	50	0.75
	10	50	1.5
	10	50	2
	10	50	3
Physiologic	5	50	/

**Fig. 2:**
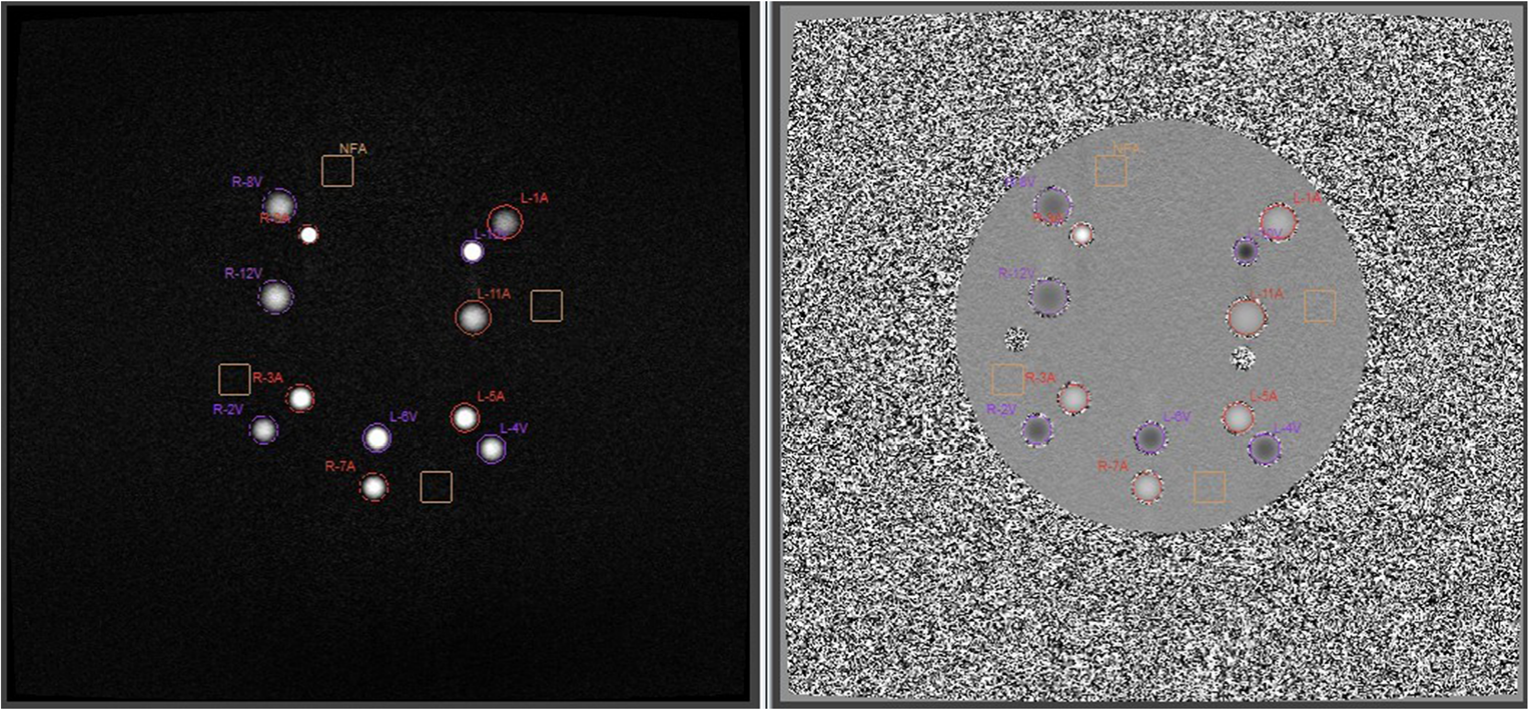
Example of magnitude (left) and phase (right) images of the phantom. Superimposition of the regions of interest of the pipes (ROIs, circles) and no flow area regions (NFAs, squares) are also reported

Each 2D acquisition was set up on the same axial slice and produced a complex dataset (magnitude-and-phase representation, see Fig. 2) composed of 30 frames sampling the waveform period; as such, the temporal resolution depended on the period of the incoming pulsation.

Flow values are estimated from the phase images, whereas the magnitude images are used for anatomical reference to properly assess the pipe contours through regions of interest (ROIs). All the pipes appear intense in magnitude images, while they appear bright or dark in phase images, depending on the flow direction. Indeed, the phase shift accumulated at the echo time *T*_*E*_ by a fluid element whose position *x*(*t*) changes according to:

1 $$ x(t)={\sum}_{n=0}^{+\infty}\frac{d^nx}{dt^n}(0)\frac{t^n}{n!} $$

under the action of an arbitrary flow-encoding gradient pulse **G**(t) is given by:

2 $$ \upphi =\upgamma {\int}_0^{T_E}\mathbf{G}(t)\cdotp x(t) dt=\upgamma {\sum}_{n=0}^{+\infty}\frac{{\mathbf{m}}_{\mathrm{n}}}{n!}\cdotp \frac{d^nx}{dt^n}(0) $$

where γ is the gyromagnetic ratio of the imaged nuclear species (^1^H, in this case) and **m**_n_ represents the *n*-th moment of the gradient pulse:

3 $$ {\mathbf{m}}_{\mathrm{n}}={\int}_0^{T_E}\mathbf{G}(t){t}^n dt $$

If we consider a steady flow:

4 $$ \phi ={\phi}_0+\upgamma {\mathbf{m}}_1\cdotp {\mathbf{v}}_{\mathbf{0}} $$

where **v**_**0**_ is the flow velocity and *ϕ*_0_ accounts for a variety of phenomena not related to the flow. However, if a second acquisition with reversed gradient $-\overrightarrow{G}(t)$ is collected, the phase difference between the two images is given by:

5 $$ \varDelta \phi =2\gamma {\mathbf{v}}_0\cdotp {\mathbf{m}}_{\mathbf{1}} $$

Therefore, it is possible to derive the velocity component parallel to **m**_**1**_ as:

6 $$ {v}_0=\frac{\varDelta \phi}{2\gamma \left|{\mathbf{m}}_1\right|} $$

It also follows from Eq. (6) that aliasing occurs when the speed exceeds the velocity-encoding (VENC) parameter, which is defined as:

7 $$ \mathrm{VENC}=\frac{\uppi}{2\gamma \left|{\mathbf{m}}_1\right|} $$

A complete list of symbols used in the above equations is reported in Table 3.

**Table 3:** List of symbols used in the equations

Symbol	Description
*x*(*t*)	Fluid element position
*G*(*t*)	Flow-encoding gradient pulse
*T*_*E*_	Echo time
*γ*	Gyromagnetic ratio of the imaged nuclear species
*ϕ*	Accumulated phase
*m*_*n*_	n-th moment of the flow-encoding gradient pulse
*ϕ*_0_	Phase not related to flow
*VENC*	Velocity-encoding parameter

We used the software package SPIN 1.5.7 (SpinTech, Inc., Bingham Farms, MI, USA) [32] to extract flow data from DICOM images. SPIN is an advanced image viewing and quantitative MR post-processing software that has been created to provide the radiologist with a tool for quantitative image analysis. Beside iron content, cerebral microbleeds, white matter hyperintensities, and perfusion weighted imaging, SPIN is particularly useful for the detection and analysis of blood flow through dedicated plugins.

The pipes were contoured on a given acquisition through an automated vessel boundary detection, which is based on region growing method with full-width half-maximum thresholding, so as to allow for consistency in inter- and intra-processing reliability. If the auto-drawn boundaries were not adequate, they were manually fixed by an expert operator and then used for the remaining acquisitions.

Inherent phase shift due to eddy currents and transient effects were removed by selecting four regions in the stationary soft-tissue mimicking liquid, which were used as phase reference for no-flow areas (NFAs).

Since **G**(t) and, hence, **m**_**1**_ were perpendicular to the imaged slice, the flow rates were derived by integrating the velocity estimates on the pipe cross sections.

## Results

Since the pipes were connected in series, we expected the same flow volumes per pulse period in the odd-labeled pipes (contoured by red ROIs in Fig. 2) and opposite values in the even-labeled pipes (contoured by purple ROIs in Fig. 2). No significant mismatch was actually found in the magnitude of the measured flows (Wilcoxon signed-rank test *p*-value of .950), thus excluding apparent inaccuracies in the pipe contours or in the choice of the NFAs.

### Constant flow

**Fig. 3:**
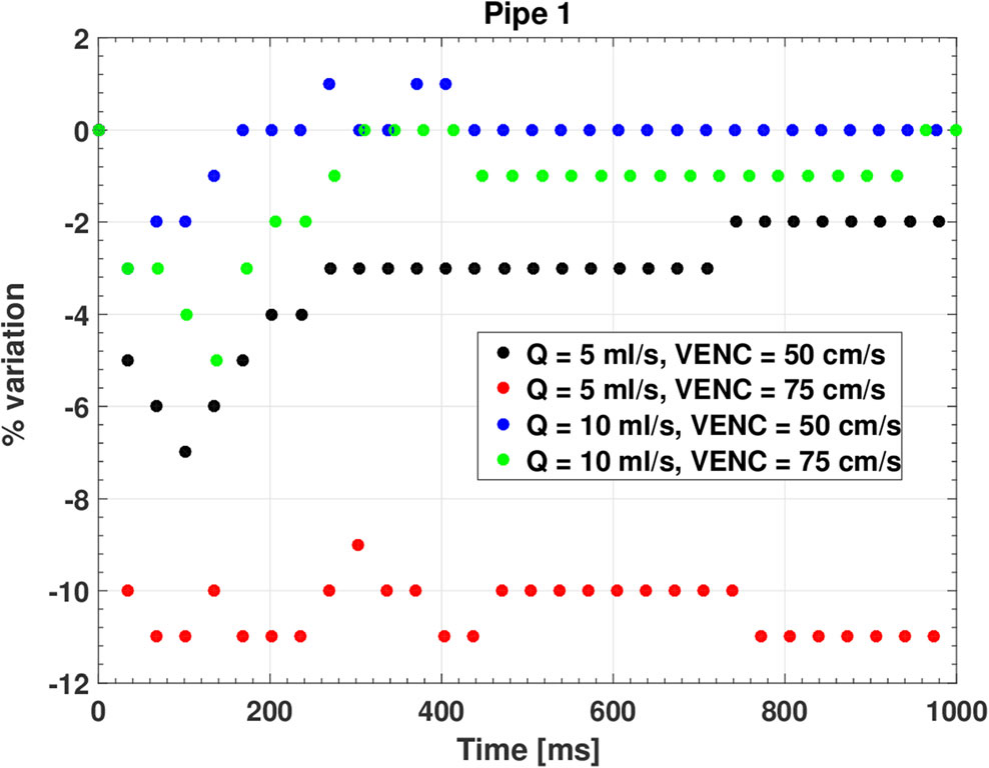
Frame-by-frame measurement of continuous flows in the first pipe (L-1 A in Fig. 2)

The frame-wise analysis of the percentage deviation of the measured flows in the first pipe (L-1 A in Fig. 2) with respect to the values set through the calibrated pump showed a good consistency of the system (Fig. 3). This is confirmed by the mismatches averaged over the acquisition period (Table 4), which are of the order of few percentage points, except for low flow (5 ml/s) measured with a high VENC value (75 cm/s).

**Table 4:** Percentage error averaged over the acquisition period of 1 s for continuous flows

VENC [cm/s]		
Flow [ml/s]	50	75
5	− 3.0 *±* 1.0	− 10.4 *±* 0.4
10	− 0.1 *±* 0.7	− 1.0 *±* 1.0

**Fig. 4:**
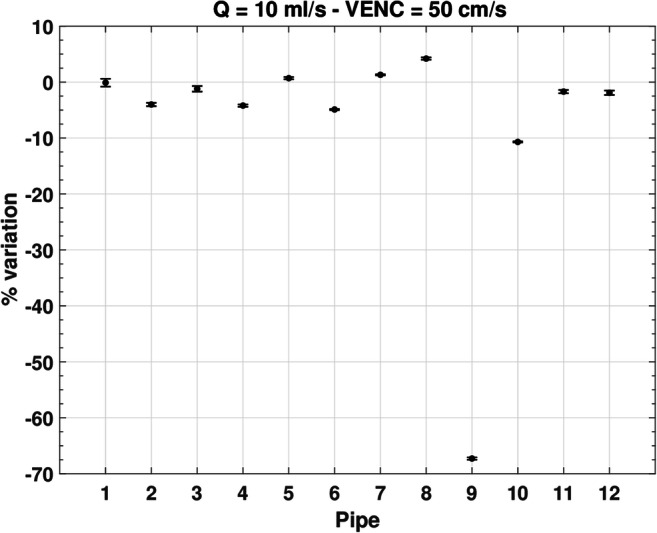
Pipe-by-pipe measurement of net flow over one period (30 frames, step size 33.6 ms)

**Fig. 5:**
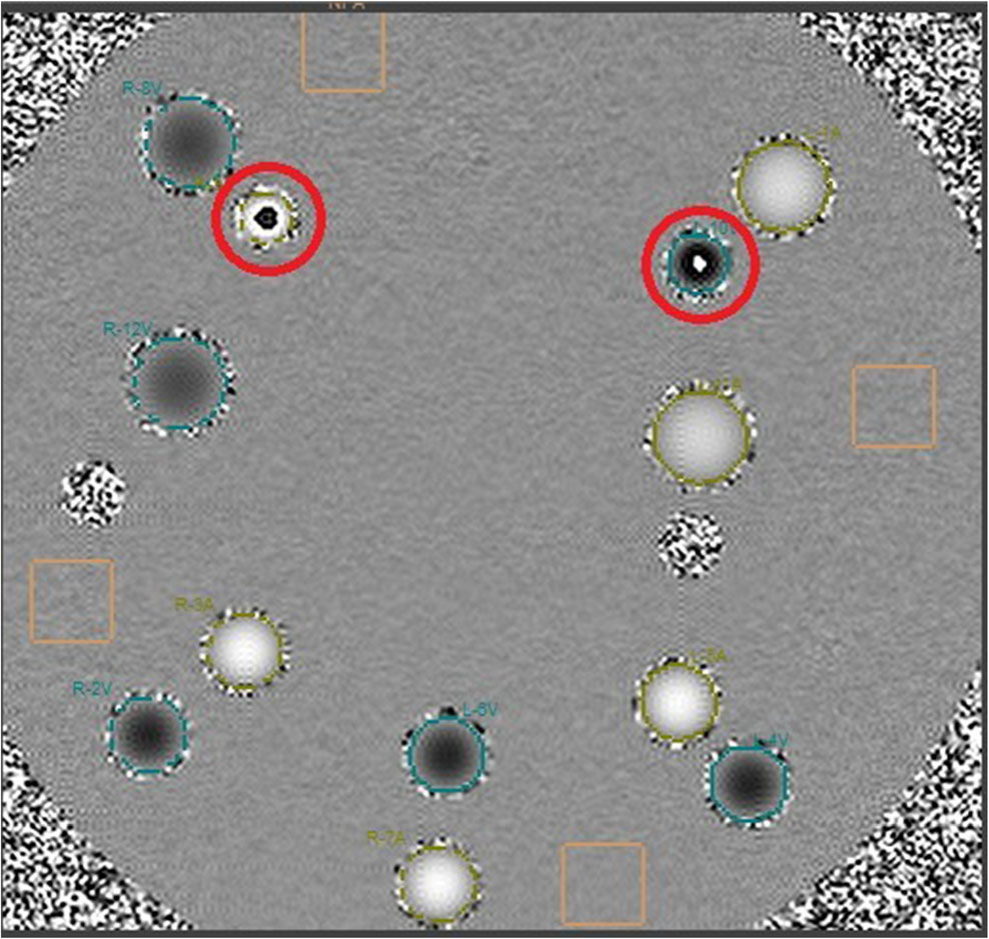
MR phase image of the phantom. Pipe 9 (left red circle) and 10 (right red circle) suffer aliasing at the level of longitudinal axis

Among all the performed tests, the optimum configuration is with the calibrated pump set at 10 ml/s and the VENC at 50 cm/s. We performed the pipe-by-pipe measurement of net flow over one period in such configuration. As shown in Fig. 4, the percentage variation from the exact value is negligible in pipe 1, and in general less than 5%, except for pipes 9 and 10, in which some aliasing occurs (Fig. 5).

### Pulsatile flow: sine flow

**Fig. 6:**
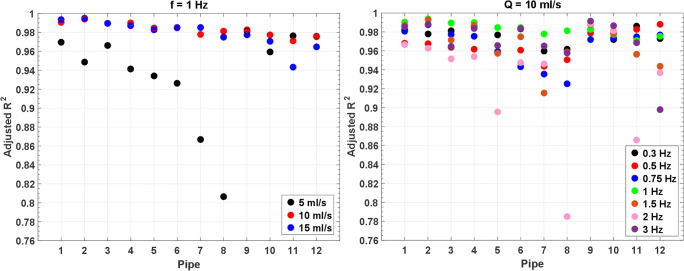
Adjusted *R*^2^ for sinusoidal flows at f = 1 Hz (left) and Q = 10 ml/s (right)

The adjusted *R*^2^ of the linear regressions concerning the frame-wise expected flow rates vs the measured ones was plotted for sinusoidal flows grouped for frequency (1 Hz, Fig. 6, left) and amplitude (10 ml/s, Fig. 6, right).

An optimal match between expected and measured flows is found for large wave amplitude (Q ≥ 10 ml/s) or low frequency (f ≤ 1 Hz), with mean adjusted *R*^2^ = 0.98 ± 0.01. Overall, the average adjusted *R*^2^ is 0.96 ± 0.02, which shows that the phantom is able to properly carry the expected sine waveform through all pipes in the analyzed range, almost regardless of the chosen frequency and amplitude. Few exceptions are found for low flow (Q = 5 ml/s) and high frequency (f = 2 Hz): in the middle-far part of the phantom (pipes 7 and 8 in Fig. 6, left, and pipes 5, 8, and 11 in Fig. 6, right), the adjusted *R*^2^ value drops below 0.90.

**Fig. 7:**
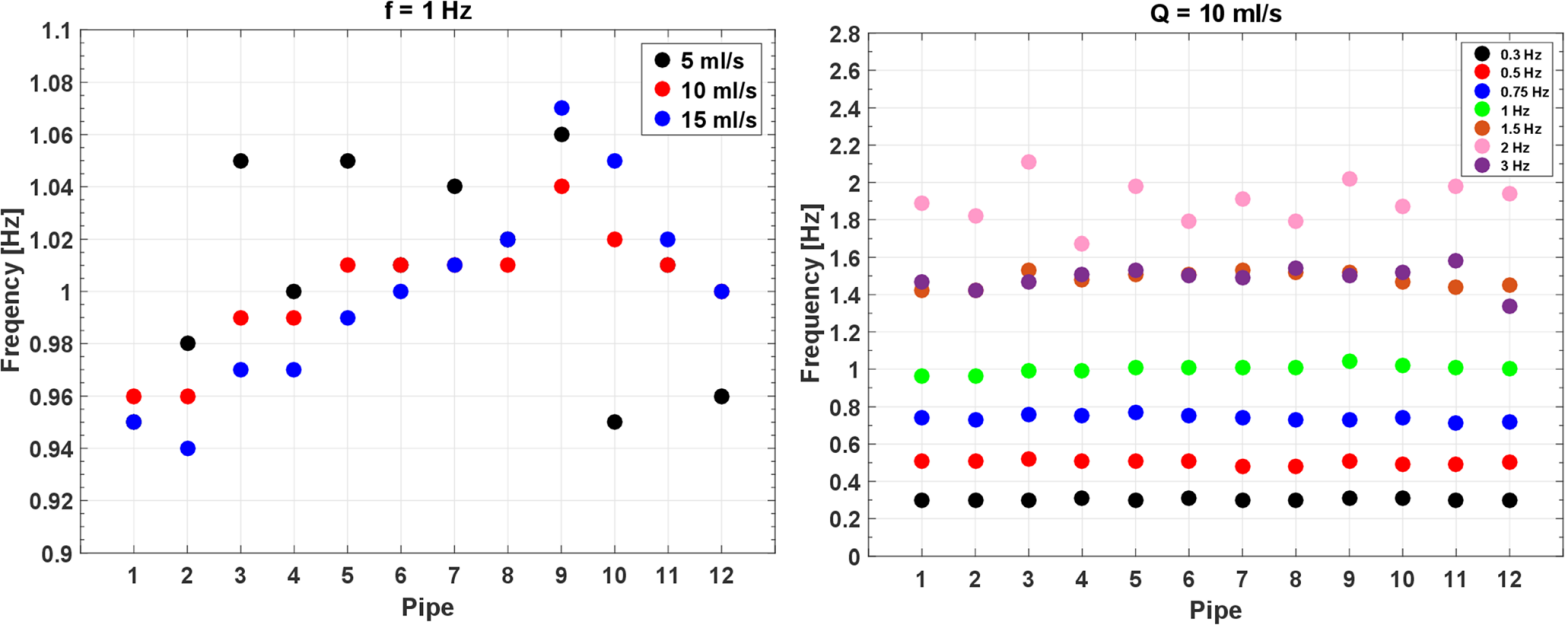
Measured frequency for sinusoidal flows at f = 1 Hz (left) and Q = 10 ml/s (right)

**Fig. 8:**
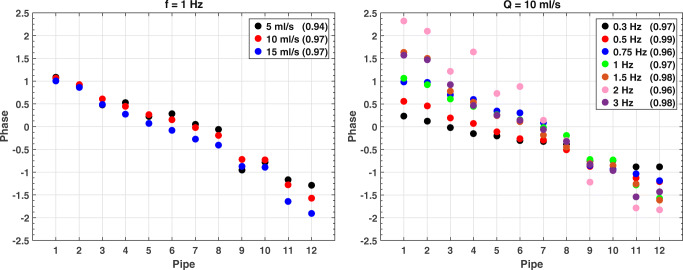
Measured phase for sinusoidal flows at f = 1 Hz (left) and Q = 10 ml/s (right). Adjusted *R*^2^ for every flow is also reported

The frequency of the fitted measurements for sinusoidal flows at f = 1 Hz (Fig. 7, left) and Q = 10 ml/s (Fig. 7, right) confirms that the selected frequency is properly measured for any tested flow (with mismatches of few percent) up to an expected frequency of ≈ 1.5 Hz. Above this threshold, large mismatches may occur between the selected frequency and the measured one. On the other hand, a clear trend can be observed between the phase of the fitted measurements and the pipe number (Fig. 8, left (mean Spearman *ρ*^2^ = 0.99 ± 0.02, *p*-value < .001) and Fig. 8, right (mean Spearman *ρ*^2^ = 0.99 ± 0.01, *p*-value < .001)). Besides, the slope is proportional (mean Spearman *ρ*^2^ = 0.93, *p*-value < .001) to the selected frequency of the waveform (Fig. 8, right). This behavior is expected since the present configuration of the phantom includes some compliant tubes connecting the glass pipes that limit the phase velocity of the waves.

**Fig. 9:**
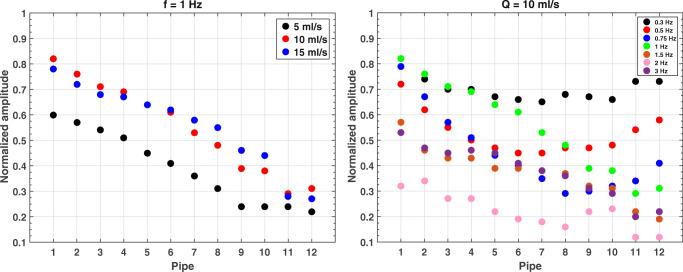
Normalized amplitude for sinusoidal flows at f = 1 Hz (left) and Q = 10 ml/s (right)

We also normalized the amplitudes of the fitted measurements to the expected values at the exit of the pumping system for sinusoidal flows at f = 1 Hz (Fig. 9, left) and Q = 10 ml/s (Fig. 9, right). The plots show that the normalized amplitudes decrease at increasing distance from the pump (mean Spearman *ρ*^2^ = 0.99 ± 0.01, *p*-value < .001 in Fig. 9 , left, and mean Spearman *ρ*^2^ = 0.63 ± 0.37, *p*-values ranging from < .001 to .344 in Fig. 9, right). Also, the measured normalized amplitude is larger for high amplitude and low-frequency sinusoidal flows. Similarly to what happens in the phase propagation, this is due to the compliant tubes that dump the selected oscillations.

### Pulsatile flow: physiologic flow

**Fig. 10:**
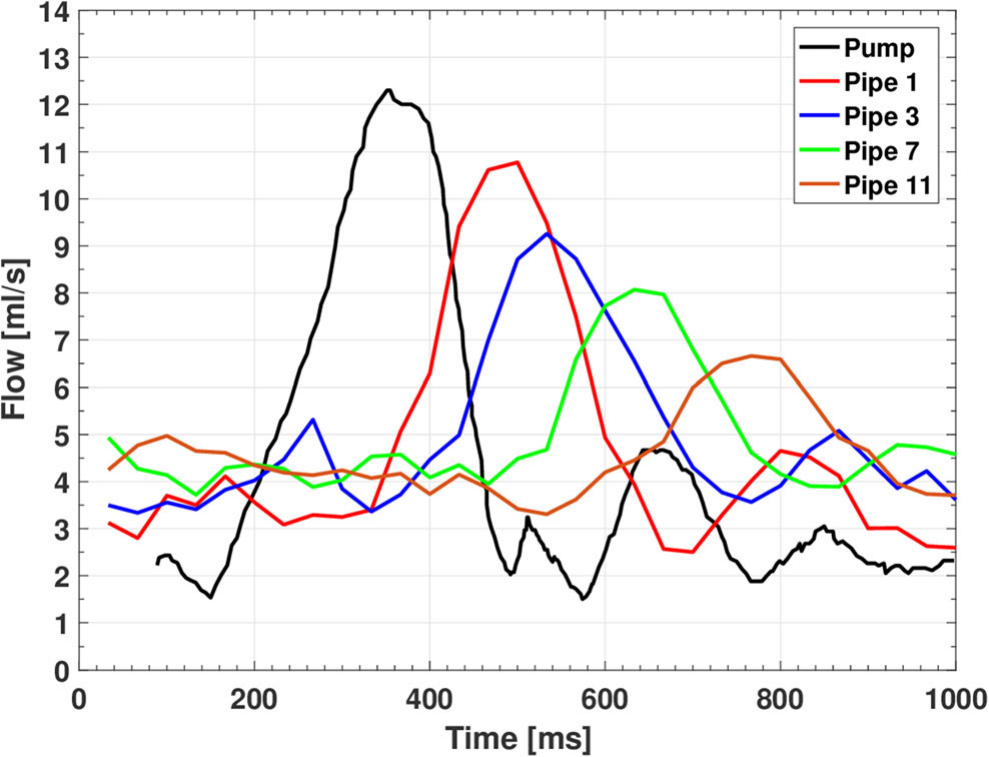
Measured physiologic carotid flow

Similar transmission patterns of the selected waveform are found when a physiologic carotid flow [33] is mimicked with the pumping system (Fig. 10). In particular, the selected carotid flow is properly recorded by the MRI scan in all glass pipes. However, similar to the sine flows, the peaks of the measured waveforms decrease in amplitude and shift in phase as the pipe number increases.

## Conclusion

In the present study, we have shown the qualitative and quantitative robustness of the NO-HYPE phantom, in compliance with the specific requested design criteria [2].

The use of quantitative MR techniques is mandatory to increase the effectiveness of qualitative MRI in medicine [34, 35]. Nevertheless, quantitative techniques strongly rely on the MRI scanner performances, thus the need of standard calibration objects [2]. The evaluation of an MRI protocol for measuring blood flow is an open and active field of research, and although many advances in MRI for flow quantification have been reached, there is still need for calibration tools [36, 37]. Indeed, some groups validated the flow patterns measured by high-resolution, time-resolved, three-dimensional PC-MRI in a real size intracranial aneurysm phantom [38], while others used a phantom to reveal that, in case of stenosis, the most accurate measures of flow by PC-MRI are found at the narrowest vessel cross section [39]. Nonetheless, it is worth to mention that these are examples of phantoms for PC-MRI flow assessment tailored on specific conditions, which cannot therefore provide an accurate and reliable estimation of the basic performances of the scanners.

Results from Fig. 6 to Fig. 7 demonstrate that the NO-HYPE phantom proposed in this study is a valid tool for the analysis of any baseline offset error, which adds an unknown offset to the measured velocities. In fact, for accurate flow measurements, this offset must be shown negligible or corrected [40]. The reliability of the NO-HYPE phantom guarantees an accurate detection of this kind of error sources. In general, comparison of the reported results against literature [2, 41] demonstrates that the phantom is a robust standardized test object useful for the evaluation of MR flow measurements.

Application-specific phantoms are largely used in clinical practice. However, they could be difficult to interface with different settings. For quantitative imaging to reach its full potential, it is necessary to analyze measurements across systems [2]. Clinical use of quantitative imaging can be facilitated through adoption and use of a standard system phantom, a calibration/standard reference object, to assess the performance of an MRI machine, or to compare and evaluate new systems for vascular monitoring [42, 43]. The presented phantom ensures quantitative MR measurement comparable over time. Moreover, its simple structure and interface with the clinical system allows it to be easily used in every MR site and with every MR machine avoiding complicated pre-settings. To date, the most extensive studies about PC-MRI have used static tissue phantoms [2]. Static tissue phantoms can be used to study phase offset errors that have large effects on the accuracy of spatially and temporally integrated phase-contrast flow measurements. The most comprehensive phantom consisted of 10- to 15-l tanks of aqueous gelatin solution, which were doped with 5 mmol/l of gadolinium-diethylenetriamine pentaacetic acid to facilitate the measurement of small background phase offsets [44]. The design of the NO-HYPE phantom is promising for studies about phase offset errors with a significant reduction of the phantom volume. There is also the need for a robust, dynamic phantom to replicate spatially and temporally varying velocities across a large range of magnitudes. In single-center, in-house studies, dynamic fluid phantoms were used to replicate pulsatile flows [45]. From this point of view, the benefit of the proposed phantom relies in the fact that it has 12 pipes that allow to check flow differences in time and space, giving room to studies of up to 12 different simultaneous pulsations. There are also studies about numerical phantoms to mimick stenotic geometries [46]. From this point of view, the benefit of the proposed phantom configuration relies in the fact that it potentially allows for simultaneous in vitro simulations of stenosis in different vessels, and/or patient specific situations, avoiding numerical simulations.

Merits of the proposed design include the compatibility with a large fraction of coil geometry, the robustness and the long-term stability of the materials, and, last but not least, the production cost commensurate with existing phantoms. Beside calibration purposes, the proposed design of phantom can be used to assess the feasibility of an MR scan to evaluate a specific biomarker and to allow for periodic quality assessment tests [45–48]. Given these features, the NO-HYPE phantom could be therefore used not only for single-institution purposes, but also in the framework of multicenter clinical trials, particularly in the first steps of the study (namely, the protocol standardization and image quality assessment).

1.4 *Design constraints and future modifications*

It should be acknowledged that the present study shows a prototypical object, which obviously suffers some drawbacks. In particular, the compliance of the connections between pipes leads to an undesired dumping of the propagated waveform. This could induce a detrimental decline of the gold standard reliability of the expected flow in the farthest pipes. Also, the relaxation properties of the BMF can be further refined in order to provide phantom images comparable to clinical acquisitions. Despite these limitations, the proposed NO-

HYPE phantom offers a straightforward and cost effective approach to test the quality of PC acquisitions in clinical scanners, with a valuable impact in the estimation of flows by means of MRI. Furthermore, since the NO-HYPE phantom flows have been validated against PC-MRI measurements, the designed tool could be used for ultrasound imaging assessment by replacing the current BMF with a fluid enhancing the Doppler response [31].

## Abbreviations

BMF: Blood-mimicking fluid
DICOM: Digital Imaging and Communications in Medicine MRI Magnetic resonance imaging
NFA: No-flow area
PC: Phase contrast
PVC: PolyVinyl chloride
QIBA: Quantitative Imaging Biomarkers Alliance
ROI: Region of interest
RSNA: Radiological Society of North America
SNR: Signal-to-noise ratio
VENC: Velocity encoding
WSS: Wall shear stress
